# Equity implications of tobacco taxation: results from WHO FCTC investment cases

**DOI:** 10.1136/tc-2023-058338

**Published:** 2024-05-02

**Authors:** Garrison Spencer, Rachel Nugent, Nathan Mann, Brian Hutchinson, Carrie Ngongo, Dudley Tarlton, Roy Small, Douglas Webb

**Affiliations:** 1 RTI International, Research Triangle Park, North Carolina, USA; 2 Department of Global Health, University of Washington, Seattle, Washington, USA; 3 United Nations Development Programme, Istanbul, Turkey; 4 HIV, Health and Development Group, United Nations Development Programme, New York, New York, USA; 5 United Nations Development Programme, Amman, Jordan

**Keywords:** Advocacy, Economics, Low/Middle income country, Disparities, Socioeconomic status

## Abstract

**Background:**

Across time, geographies and country income levels, smoking prevalence is highest among people with lower incomes. Smoking causes further impoverishment of those on the lower end of the income spectrum through expenditure on tobacco and greater risk of ill health.

**Methods:**

This paper summarises the results of investment case equity analyses for 19 countries, presenting the effects of increased taxation on smoking prevalence, health and expenditures. We disaggregate the number of people who smoke, smoking-attributable mortality and cigarette expenditures using smoking prevalence data by income quintile. A uniform 30% increase in price was applied across countries. We estimated the effects of the price increase on smoking prevalence, mortality and cigarette expenditures.

**Results:**

In all but one country (Bhutan), a one-time 30% increase in price would reduce smoking prevalence by the largest percent among the poorest 20% of the population. All income groups in all countries would spend more on cigarettes with a 30% increase in price. However, the poorest 20% would pay an average of 12% of the additional money spent.

**Conclusions:**

Our results confirm that health benefits from increases in price through taxation are pro-poor. Even in countries where smoking prevalence is higher among wealthier groups, increasing prices can still be pro-poor due to variable responsiveness to higher prices. The costs associated with higher smoking prevalence among the poor, together with often limited access to healthcare services and displaced spending on basic needs, result in health inequality and perpetuate the cycle of poverty.

**WHAT IS ALREADY KNOWN ON THIS TOPIC**

- People with low incomes are more likely than wealthier individuals to use tobacco and are also more responsive to changes in the price of tobacco.

**WHAT THIS STUDY ADDS**

- We report the results of equity analyses conducted as part of 19 national tobacco control investment cases, demonstrating the pro-poor effects of increased tobacco taxation in terms of tobacco-attributable mortality and expenditures across diverse national contexts.

**HOW THIS STUDY MIGHT AFFECT RESEARCH, PRACTICE OR POLICY**

- The investment case process in multiple countries has demonstrated the salience of equity analyses in advocating for tobacco control, especially increased taxation, among diverse stakeholders across sectors.

## Introduction

The WHO Framework Convention on Tobacco Control (FCTC) 2030 project was implemented by the WHO FCTC Secretariat and the United Nations Development Programme along with RTI International and other technical partners.^1^ The programme has supported low and middle-income countries (LMICs) to conduct investment cases examining the benefits and costs of a set of tobacco control interventions.^2^ Investment cases included a customised analysis on a topic of the country’s choosing. Countries often selected to receive an equity analysis examining the differential impact of increases in tobacco taxes on socioeconomic groups. In choosing a topic for customised analysis, stakeholders selected what type of results would be most effective in advancing the tobacco control agenda in their national context. That an equity analysis was the most selected custom analysis demonstrates the commitment to the 2030 Agenda for Sustainable Development pledge to leave no one behind and reach the furthest behind first.^3^

Across time, geographies and country income levels, there is a robust association between lower incomes and higher smoking prevalence.^4^ Tobacco use both exacerbates and is exacerbated by poverty.^5^ The poor may be less aware of the harms of tobacco use and particularly vulnerable to tobacco industry marketing tactics due to lower levels of education and literacy. Some people may turn to tobacco as a coping mechanism for the many stresses of living in poverty. At the same time, the price of a pack of cigarettes represents a larger share of disposable income among the poor compared with wealthier individuals and may displace spending on education, food or healthcare. When a head of household falls ill due to tobacco-attributable diseases, the loss of income can be especially devastating for those living in poverty. Indeed, households with at least one person who smokes are more likely to experience catastrophic health expenditures (defined as out-of-pocket healthcare expenses more than 40% of a household’s capacity to pay).^6^ It is therefore especially important that tobacco control efforts effectively reach the poor. If interventions do not reach or are less effective among the poor than among wealthier income groups, the resulting inequity imposes a dual burden, as they already shoulder a disproportionate share of the health and economic costs of tobacco use.^5^

A systematic review of the effect of tobacco control interventions on socioeconomic inequalities that included price increases, smoke-free environments, education and media campaigns, and cessation support found that price increases were the only policy to have a pro-equity effect and that others were ‘unlikely to reduce inequalities in smoking without specific efforts to reach disadvantaged smokers’.^7^ Taxes have been widely applied to discourage consumption of unhealthy products, such as alcohol, sugar-sweetened beverages and tobacco.^8^ The equity implications of such taxes have also been widely studied. While there are exceptions, the literature suggests a strong relationship between income and responsiveness to changes in price. This means that when prices increase, the poor reduce their consumption at greater rates than wealthier individuals.

This paper summarises the results of investment case equity analyses for 19 countries and the implications for sustainable development, presenting the effects of increased taxation on smoking prevalence, health outcomes and expenditures.

## Methods

National health authorities in 19 countries chose to include analyses investigating the equity implications of cigarette taxation in their national investment cases through the WHO FCTC 2030 project. This section describes the methods of these equity analyses and updates made to improve comparability in this cross-country presentation of results. Full investment case reports for most of the countries included in this analysis are available on the WHO FCTC website; however, several remain under review and are not yet publicly available.^9^

We performed a simulation study of the effect of a standardised increase in price of cigarettes using smoking prevalence data by quintile reported by country surveys and standard quintile elasticities of demand reported in the literature. Our outcomes of interest were changes in smoking prevalence, smoking-attributable mortality and expenditures on cigarettes to demonstrate the distributional effects of increases in cigarette taxes. More complex studies have been published that examine additional outcomes, such as changes in healthcare expenditures and financial risk protection, and provide further evidence of the pro-poor nature of tobacco taxation.^10–12^ For example, in China, a 50% increase in the price of tobacco was estimated to decrease healthcare expenditures by US$21 billion and US$1.8 billion in financial risk protection with 28% and 74% of savings benefiting the lowest income quintile, respectively.^11^ To meet the needs and pace of the WHO FCTC 2030 project, however, simplicity and the ability to produce estimates in a timely manner were prioritised to be able to incorporate these equity analyses in many of the investment cases.

We sought smoking prevalence data disaggregated by income quintile: that is, five groups each containing 20% of the population. The first quintile contains the poorest 20% of the population and the fifth quintile contains the wealthiest 20%. Income quintile-specific smoking prevalence data for 12 countries were available from national Demographic and Health Surveys (DHS) (Burkina Faso, Cambodia, Chad, Egypt, Ghana, Myanmar, Nepal, Pakistan, Sierra Leone, Tanzania, Timor-Leste and Vanuatu).^13–24^ Other national-level surveys also served as sources, including WHO STEPwise Approach to NCD Risk Factor Surveillance surveys (Mozambique and Bhutan), Multiple Indicator Cluster Surveys (Eswatini, Laos and Samoa) and the Adult Tobacco Consumption Report and the Health Examination Survey in Serbia and Tunisia, respectively. Surveys were conducted between 2010 and 2019.^25–31^

We used surveys with data for various age groups: 15 and older, 15–49, 15–69 and 18–85. Where possible, we used manufactured cigarette smoking prevalence (available in 12 of 19 countries); however, surveys in six countries only reported smoking prevalence rates for all types of tobacco products or combined manufactured cigarette smoking and another type of cigarettes (eg, clove cigarettes, or ‘kreteks’). Additionally, the assessment of current smoking prevalence varied slightly between survey instruments. For example, the standard DHS survey instrument asks respondents if they have smoked any tobacco product in the past 30 days and if they smoke cigarettes every day, some days or not at all, while the standard WHO STEPwise Survey instrument asks only if respondents are current smokers and, if so, if they smoke every day.^32 33^ These differences in age groups, tobacco products and use definitions limit direct comparability. To estimate the number of people who smoke in each income quintile, we multiplied quintile-specific prevalence by one-fifth of the total population of the age group specified from the source for smoking prevalence.^34^ In countries with survey data for ages that did not include the total adult population, for example, ages 15–49, the impacts for ages 50 and above were not included.

We modelled a scenario in which a uniform 30% increase in the price of the most sold brand of cigarettes was applied across all countries. A 30% increase was selected because it is comparable to, or more conservative than, increases modelled in other studies or those implemented in practice in countries around the world.^10 11 35^ The price of cigarettes in each country was extracted from the appendix of the 2021 WHO Report on the Global Tobacco Epidemic, which contains data on the price of a 20-cigarette pack of the most sold brand in each country.^36^ This assumes that the most sold brand of cigarettes is representative of the market and does not incorporate other market segments (low-end or high-end cigarettes). Models that account for switching between market segments in response to changes in price would capture nuance helpful to framing tobacco tax policy and estimating impact; however, market segment data were not available across countries. Even when cheaper cigarettes are available, studies show that higher cigarette taxes still prevent people from starting to smoke, encourage cessation and reduce smoking intensity; and that uniform specific excise taxes reduce price differences between brands, which in turn limits the possibility for switching in response to increased taxes.^37^ Price elasticity of demand is the degree to which one variable changes in response to changes in another variable and is the key economic concept of this analysis—in this case, the degree to which cigarette consumption responds to changes in price. Income-specific price elasticity of demand was derived from Fuchs *et al*, who estimated the price elasticity of tobacco demand for seven LMICs (Bangladesh, Bosnia and Herzegovina, Indonesia, Moldova, Russia, South Africa and Ukraine) and one high-income country (Chile) by income decile.^38^ All eight countries showed that elasticity decreases as income rises. Because our prevalence data were available by quintiles (not deciles), we took the average of two deciles to estimate quintile elasticity. We then derived the simple average from the eight countries to apply to all 19 countries of our analysis.

The price elasticity of demand indicates the effect of increases in price on overall consumption, both from reductions in smoking intensity (people smoking less) as well as in smoking prevalence (people quitting smoking or not starting to smoke). For our analysis, we were interested only in the effect on prevalence (the number of people who smoke) and not reduced consumption (the number of cigarettes smoked). This is because prevalence captures only those who quit smoking or do not initiate smoking and not those who reduce their consumption. The harms of smoking occur at any level of consumption.^39^ While risk of smoking-attributable diseases falls somewhat due to reduced consumption, due to data limitations we could only include health benefits from quitting tobacco use accruing to those who quit. This results in a conservative estimate of the benefits of increased taxation as the reduced risk resulting from reduced consumption (rather than cessation) was not captured. Following Goodchild *et al*, we assumed that half of the reduction in overall consumption comes from reduced consumption in those who continue smoking, and the other half from cessation or prevented smoking initiation.^40^ The price elasticity of demand and prevalence elasticity (the change in smoking prevalence, rather than consumption, resulting from changes in price) used in the analysis are provided in table 1.

**Table 1:** Elasticity of demand and elasticity of prevalence, by income quintile

	Quintile 1	Quintile 2	Quintile 3	Quintile 4	Quintile 5
Elasticity of demand	−0.60	−0.49	−0.41	−0.36	−0.30
Elasticity of prevalence	−0.30	−0.24	−0.21	−0.18	−0.15

We chose to apply the elasticities to all countries because country-specific estimates of income group-specific elasticities are not available for the majority of countries in our study. Income group-specific estimates from Myanmar, Nepal and Egypt rely on data that are over two decades old and may not accurately reflect more recent consumption behaviour.^41–43^ More recent estimates from Serbia are only available for low, middle and high-income groups (rather than by quintile) but show a similar trend with elasticities of demand of −0.51, –0.37 and −0.22, respectively.^44^ The use of these elasticities for all countries may underestimate the effect of price increases in countries with larger elasticity estimates and overestimate effects in those with smaller elasticities; as well as countries with varying distributions of elasticities between quintiles. While females consistently smoke at lower rates than males, when it comes to responsiveness to changes in price, the evidence on differences in elasticities between men and women is mixed.^45^ Therefore, we used the same elasticity for men and women and did not present sex-disaggregated results for the effects of increases in price.

The impact of changes in price on prevalence was calculated following Joossens *et al*,^46^ who used a log-log function to ensure large price increases do not result in implausible reductions in consumption or prevalence. The equation used to calculate the impact of changes in price on smoking prevalence in each quintile is:

$\Delta S_{i}={S_{i-1}}^{*}((EXP(\varepsilon p^{*}LN(r))$

where *S_i_* is the number of people who smoke in year *i*, *Ԑp* is prevalence elasticity and *r* is the ratio of the old price of a pack of cigarettes to the new price after the increase. Because we modelled a one-time increase in the price of cigarettes, we did not include the impact of changes in individuals’ income on consumption (income elasticity).

Smoking-attributable mortality data were downloaded from the Global Burden of Disease (GBD) study website, which contains estimates of the extent to which smoking contributes to the incidence of 37 diseases and deaths across 195 countries.^47^ To calculate the reductions in mortality due to the increase in price, the estimated change in smoking prevalence was applied directly to the GBD smoking-attributable deaths.

To calculate the amount spent on cigarettes, we first needed to know the number of cigarette packs sold in a country—a figure which was not always readily available. Instead, we estimated this figure with a back calculation using total tax revenue and tax amount per pack of the most sold brand of cigarettes, both of which were available in the appendices of the WHO Report on the Global Tobacco Epidemic.^36^ One limitation of this method is that the total tax revenue reported by countries is sometimes inclusive of all tobacco products, not only cigarettes, resulting in an overestimation of the number of cigarette packs sold. We assumed that our estimated number of packs consumed is distributed in the same proportion as the number of people who smoke in each income quintile. This assumption implies that all people who smoke consume the same number of cigarettes per day regardless of income level or sex. While there may be differences in smoking intensity (number of cigarettes smoked per day) between socioeconomic groups across countries to varying degrees, previous analysis of household survey data from over 80 LMICs found that manufactured cigarette smoking intensity was relatively similar across income quintiles.^48^

Some data have been updated if more recent data have become available since the completion of the investment case (such as prevalence data by income quintile from a newer survey), or to increase the comparability between countries (such as applying a uniform price increase across countries). This may result in slight changes to the results that were previously included in individual country investment cases.

## Results

### Current burden

Smoking patterns between sexes and income groups vary greatly between the 19 countries of the analysis (table 2). In 14 of the countries (74%), overall smoking prevalence was greater in quintile 1 than in quintile 5. The quintile with the highest smoking prevalence is denoted by bold values in table 2. In seven countries, the prevalence in quintile 1 was more than double the prevalence in quintile 5 (Ghana, Cambodia, Sierra Leone, Mozambique, Chad, Laos and Nepal). In absolute terms, the largest gap was among males in Cambodia, where prevalence is 28 percentage points higher in quintile 1 than quintile 5 (46.9% and 19.0%, respectively). Overall, females smoke at much lower rates than males. On average across the 19 countries, male prevalence was 26 percentage points higher (in absolute terms) than female prevalence.

**Table 2:** Smoking prevalence by income quintile and smoking-attributable deaths

Country	Year of survey	Smoking age	Overall smoking prevalence (%)	Smoking prevalence by quintile (bold indicates the quintile with the highest smoking prevalence), %					Total smoking-attributable deaths (n), all income quintiles (2019)	Percent (%) of smoking-attributable deaths that occur among quintile 1
				1	2	3	4	5		
Bhutan	2019	15–69	10.6	4.4	7.6	9.6	11.0	**14.7**	99	9.3
Burkina Faso	2010	15–49	10.9	**11.6**	10.8	10.0	11.1	10.8	3796	21.4
Cambodia	2014	15–49	16.7	**26.2**	19.6	17.9	13.9	9.5	18 884	30.1
Chad	2015	15+	8.0	**9.7**	6.5	4.0	2.6	4.2	3839	35.9
Egypt	2017	15–59	23.6	22.8	24.4	22.6	**24.9**	23.0	96 219	19.4
Eswatini	2015	15–49	7.7	6.3	5.2	7.3	6.3	**7.8**	473	19.0
Ghana	2014	15–49	2.0	**4.6**	2.8	2.5	0.8	0.3	6773	41.8
Laos	2017	15–49	23.7	**32.3**	28.4	25.6	19.9	14.3	6982	26.8
Mozambique	2015	15–49	12.6	**16.0**	15.8	14.2	10.2	6.7	10 332	25.4
Myanmar	2015–2016	15–49	16.4	**18.2**	16.2	15.1	17.0	15.7	70 093	22.1
Nepal	2016	15–49	14.8	**22.0**	15.8	14.0	14.1	10.2	39 294	28.9
Pakistan	2017–2018	15–49	12.9	**16.5**	12.3	12.5	13.8	10.0	167 419	25.4
Samoa	2019	15–49	24.4	25.0	**26.7**	24.1	24.3	21.3	283	20.6
Serbia	2019	18–85	38.2	36.3	35.1	36.4	39.6	**43.7**	29 658	19.0
Sierra Leone	2019	15–49	10.8	**16.1**	13.5	12.1	8.5	6.1	2914	28.5
Tanzania	2015–2016	15–49	7.1	**9.8**	7.2	7.2	6.6	5.8	21 643	26.8
Timor-Leste	2016	15–49	28.5	**31.3**	30.5	28.6	26.5	26.5	1116	21.8
Tunisia	2016	15+	22.2	20.1	**24.4**	23.8	23.0	20.3	14 109	18.0
Vanuatu	2013	15–49	30.0	**34.4**	26.6	31.3	27.8	31.2	314	22.7

Source: Demographic and Health Surveys (DHS),^14–25^ WHO STEPwise Approach to NCD Risk Factor Surveillance (STEPS) surveys,^25 26^ Multiple Indicator Cluster Surveys (MICS),^27–29^ national surveys^30 31^ and the Global Burden of Disease (GBD) study.^47^

Smoking-attributable diseases, deaths and productivity losses occur at higher rates in groups with higher smoking prevalence, that is, among males and among the poor in most countries. Table 2 shows the percentage of smoking-attributable deaths that occurred in the poorest quintile of population (quintile 1). Among females in Cambodia, Myanmar and Chad, nearly half of smoking-attributable deaths occurred in quintile 1.

### Impact of price increase

**Figure 1:**
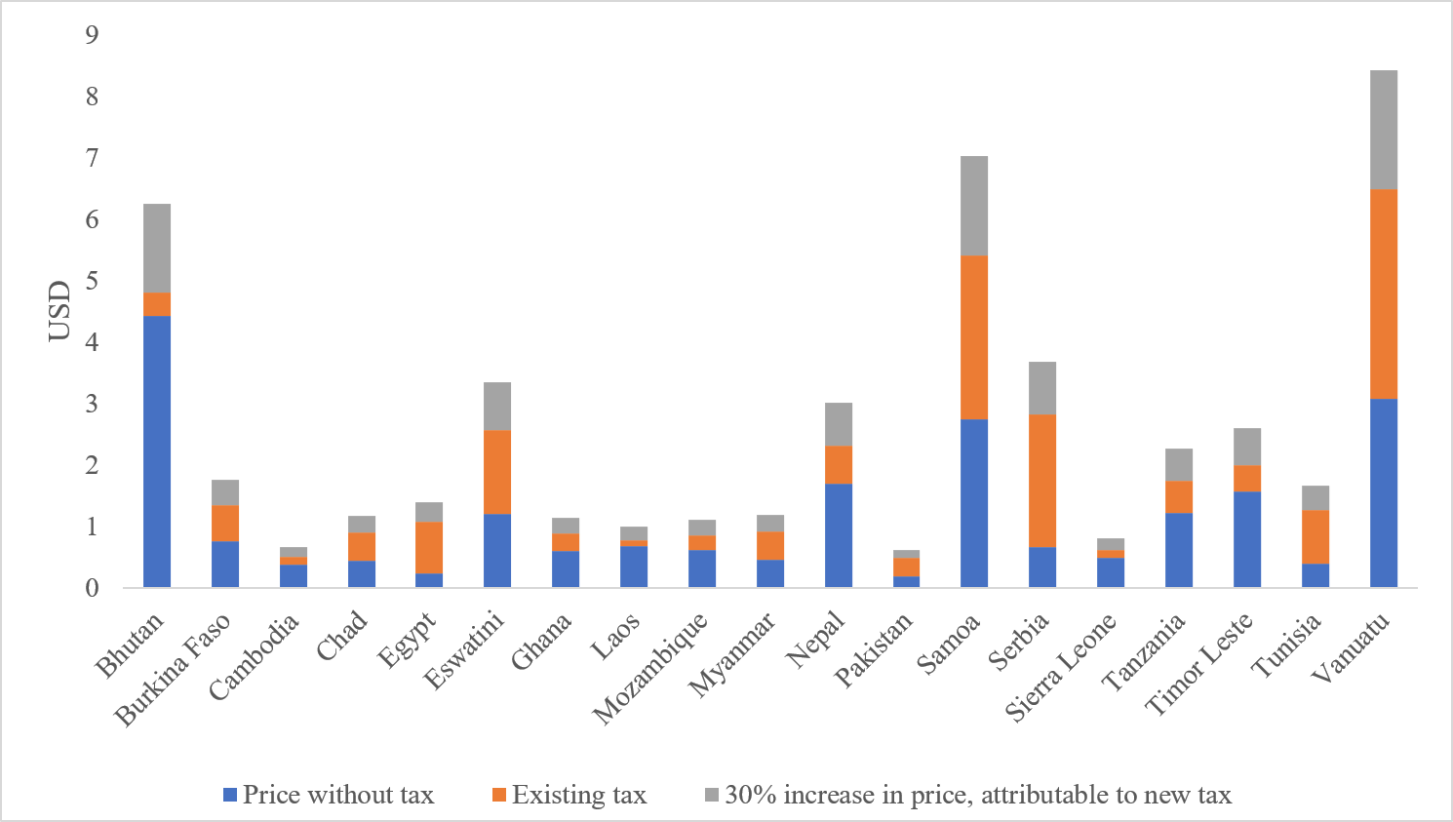
Price of most sold pack of cigarettes without tax, existing tax and 30% increase in price (attributable to new tax).

We modelled the impact of a 30% increase in the price of cigarettes on prevalence, expenditures and health outcomes. The current price of a pack of the most sold brand of cigarettes ranged from US$0.48 in Pakistan to US$6.49 in Vanuatu (table 3 and figure 1). Taxes as a percent of total price ranged from 8.1% in Bhutan to 78.5% in Egypt. Assuming that the entirety of the increase in price results from increased taxation, the price increase resulted in the largest jump in taxes as a percent of total cost in the countries with the lowest current tax rates. Tobacco companies often pass on the cost of tax increases fully to consumers and employ differential shifting, that is, increasing the price above and beyond the tax increase (overshifting) for more expensive brands and undershifting for cheaper brands in an effort to undermine tax policies.^49^ A recent systematic review of tobacco industry pricing strategies found that in LMICs, the predominant strategy was undershifting across brands, signifying efforts at market expansion rather than profit maximisation. Such undershifting would result in lower price increases than those modelled in this analysis and subsequently less health and economic benefits.^49^ Two countries (Serbia and Egypt) currently meet levels considered in the WHO Report on the Global Tobacco Epidemic 2021 as a high level of achievement, which is for total taxes to represent at least 75% of the retail price.^36^ The price increases modelled bring one additional country (Tunisia) above that threshold with a 30% increase in price.

**Table 3:** Cost of most sold brand of cigarettes (US$), cigarette tax and cost with 30% increase in price

Country	Cost of pack of cigarettes (US$)	Tax as a percent (%) of total price	Cost of pack of cigarettes with 30% increase	Tax as a percent (%) of total price with 30% increase in price
Bhutan	4.81	8.1	6.26	**29.3**
Burkina Faso	1.35	43.5	1.76	*56.5*
Cambodia	0.51	26.4	0.66	**43.4**
Chad	0.90	51.6	1.17	*62.8*
Egypt	1.07	78.5	1.39	83.5
Eswatini	2.57	53.5	3.34	*64.2*
Ghana	0.88	31.8	1.14	**47.5**
Laos	0.77	11.7	1.01	**32.0**
Mozambique	0.85	28.5	1.10	**45.0**
Myanmar	0.91	49.9	1.19	*61.5*
Nepal	2.32	27.0	3.02	**43.8**
Pakistan	0.48	60.8	0.62	*69.8*
Samoa	5.41	49.2	7.04	*60.9*
Serbia	2.83	76.5	3.68	82.0
Sierra Leone	0.62	22.6	0.80	**40.4**
Tanzania	1.74	30.0	2.26	**46.1**
Timor-Leste	2.00	21.8	2.60	**39.8**
Tunisia	1.27	69.6	1.66	76.6
Vanuatu	6.49	52.7	8.44	*63.6*

Underline denotes tax as a % of total price 70% or greater. *Italic* denotes tax as a % of total price 50–70%. **Bold** denotes tax as a % of total price less than 50%.

Source: WHO Report on the Global Tobacco Epidemic.^36^

**Figure 2:**
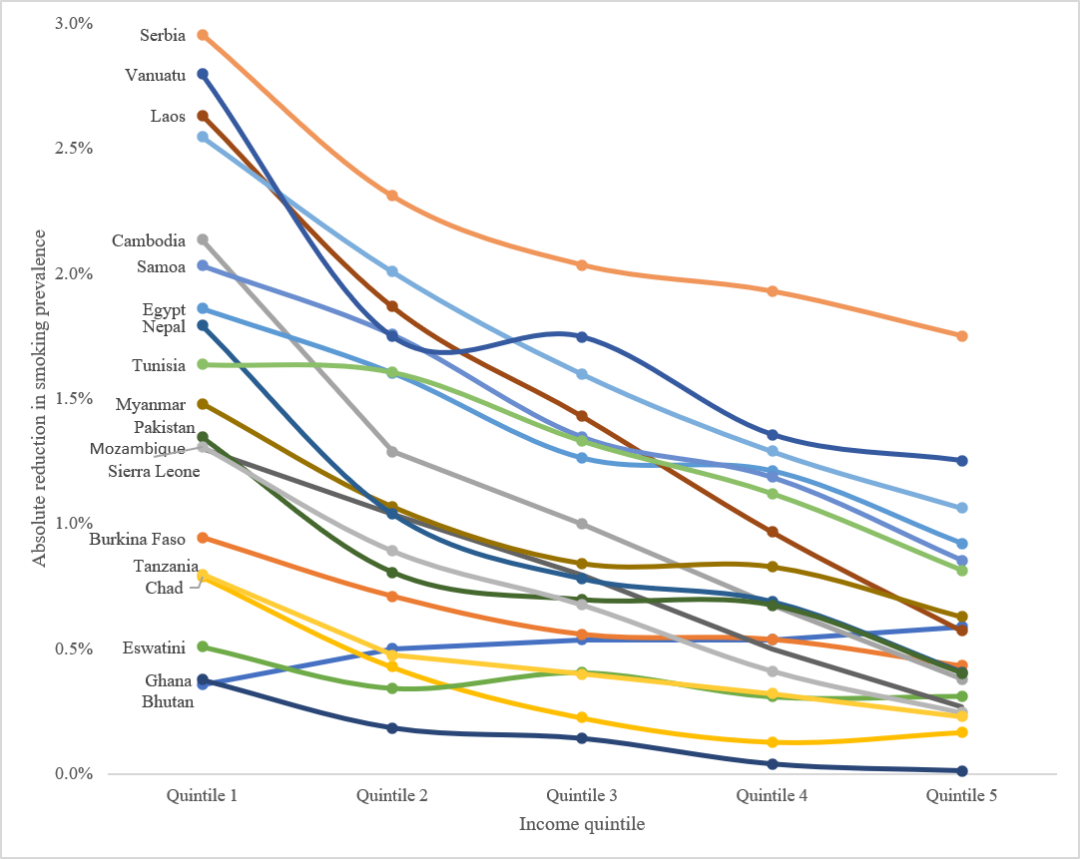
Absolute reduction in smoking prevalence resulting from 30% increase in price, by income.

The modelled 30% increase in price caused reductions in smoking prevalence among all income groups in all countries, with the largest absolute reductions seen in the countries with the highest overall smoking prevalence, such as Serbia, Vanuatu and Timor-Leste (figure 2). Because of greater responsiveness to changes in price, prevalence declined the most in quintile 1 even when wealthier groups have higher initial smoking prevalence, such as in Egypt. The poorest income quintile experienced the largest decline in smoking prevalence in every country except for Bhutan, where smoking prevalence is over three times greater in quintile 5 than in quintile 1. Bhutan represents an unusual circumstance due to a national ban on the sale of tobacco products in 2010. Bhutanese travelling abroad were allowed to import tobacco products for personal use, providing those with the means to travel abroad with much greater access to tobacco products. Bhutan has since reversed the ban on domestic sales in the wake of the COVID-19 epidemic to reduce cross-border contact.^50^

The reduced smoking prevalence resulting from a 30% increase in price would prevent over 29 600 deaths among the 19 countries over 1 year. Altogether, 33% of lives saved occurred among the poorest 20% of the population. Bhutan, where only 9% of people who smoke belong to the poorest quintile, is the only country in which quintile 1 received less than 20% of lives saved. In the other 18 countries, the poorest quintile received between 25% (Tunisia) and 50% (Ghana) of lives saved as a result of the increase in price. The price increase also disproportionately benefited the second poorest income group (quintile 2). In every country but Eswatini, quintile 2 received more than 20% of the tobacco-attributable deaths averted.

**Figure 3:**
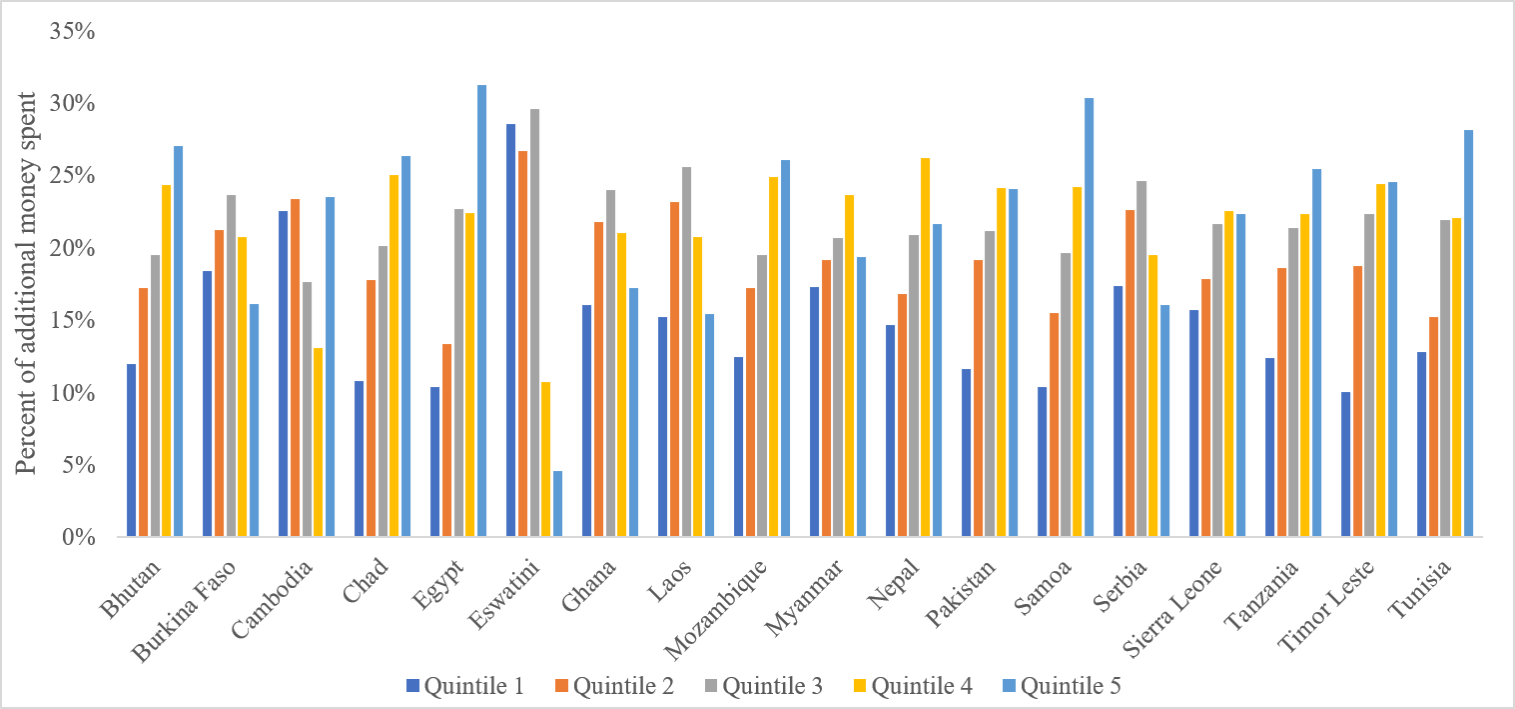
Additional expenditures resulting from 30% increase in price, distribution by quintile, in 19 countries.

Cigarettes are an inelastic good, meaning that consumption does not decrease proportionately with increases in price. This is a reflection of nicotine’s addictive nature and that there are no close substitutes that consumers can switch to when prices increase. Because of this inelasticity, the total amount of money spent on cigarettes goes up with price increases, despite the reduction in smoking prevalence. All income groups in all countries spend more on cigarettes with a 30% increase in price. However, the poorest 20% of the population pay only 12% of the additional money spent across countries (Bhutan is not included due to a lack of data to estimate consumption). Quintile 1 would pay for more than 20% of the additional money spent in Ghana (28.5%) and in Chad (22.5%), though they would pay as little as 10% of additional money spent in Eswatini, Serbia and Tunisia (figure 3).

## Discussion

By offering country-specific evidence about the distributional effects of tobacco taxation, we show the significant differences in tobacco-attributable mortality and morbidity and resulting health and economic costs that can occur among different income groups with increases in tobacco prices. The results of our analysis show that in most countries low-income populations bear a disproportionate share of the health burden resulting from smoking, and that increasing cigarette prices through taxation disproportionately benefits the poor. The economic cost of higher prevalence of smoking among the poor, together with displaced spending on basic needs and often limited access to healthcare services, results in health inequality and perpetuates the cycle of poverty. Evidence from several LMICs shows that the spending on cigarettes crowds out expenditures on education and clothing and, in low and middle-income households, on food expenditures as well.^51^ This demonstrates the high opportunity cost of tobacco use, whereby households are foregoing investments in human capital to spend money on cigarettes.

The investment cases estimate that taxation would produce the highest return on investment of all the tobacco control strategies included, as laid out by Mann *et al*, which presents the wider results of the investment cases.^52^ However, taxation should not be implemented in isolation. For those who continue to smoke after increases in price, the higher cost of cigarettes represents a larger share of poor individuals’ income compared with wealthier individuals. This contributes to continuing impoverishment and poor health among those least able to manage it. Because of this, governments have an obligation to pursue additional avenues to discourage smoking. Reinvesting tax revenue can help compensate for the negative impact that higher prices may have on low-income people who smoke but do not quit in response to increases in price. Price increases through taxation can be used to reduce inequalities if the resulting government revenue is reinvested in education, healthcare or welfare programmes. The rationale for earmarking (designating for a specific purpose) tobacco tax revenue is stronger than for other types of tax revenue because of the large cost imposed on governments from tobacco use and the ability to direct resources from those engaged in this unhealthy behaviour to fund the health sector or other public services that serve them, including health promotion and welfare programmes.^53^ Opponents of earmarking point to the potential for inefficiencies, limited flexibility and budget offsetting that may fail to increase the overall budget for health.^54^ However, earmarks may increase the political palatability of health taxes, signal government priorities and fill in gaps when existing funding is insufficient. Three of the 19 countries provide examples of how tobacco taxes can be earmarked for development priorities. In Chad, a specific excise tax of CFA100 (about US$0.20) per pack of cigarettes is applied towards universal health coverage; in Egypt, EGP0.75 (about US$0.05 at the time of writing) per pack goes towards funding national health insurance; and in Nepal, 25% of tobacco excise taxes are directed to a health tax fund which supports a cancer hospital and prevention and treatment of cancer, tuberculosis and similar diseases.^36^ Our analysis shows that the wealthiest 40% of the population (quintiles 4 and 5) pay 49% of the additional taxes collected in Nepal, 51% in Chad and 54% in Egypt, demonstrating the distributional effects of cigarette taxation and earmarking.

A recurring concern among government officials is the possibility of increased rates of tobacco taxation leading to decreased government revenue. Instead, there is an established literature showing that governments that enact substantial tax increases see increased revenue due to the increased tax per pack of cigarettes bringing in more revenue than is lost to reductions in the number of packs sold.^55^ While there must be a point where consumption drops so low that revenue would decrease, there is no evidence of a country reaching this point. Analyses on tax revenue conducted in several of the investment cases show that even aggressive increases in taxation lead to increased government revenue.^56^ Another common concern, and one often pushed by the tobacco industry, is that increasing taxation leads to increased illicit trade in cigarettes. Instead, research has shown that the underlying cause of illicit trade is weak tax administration, not the level of the tax itself.^57^

The findings of these equity analyses and the wider investment case results provide policymakers with data on the costs of tobacco use and benefits of tobacco taxation, especially for their poorest citizens, empowering them with evidence to counter some of the barriers to increased tobacco taxation. The equity analyses and investment cases can be used to position increased tobacco taxation as a sustainable, government-led financing option, demonstrate the relevance of tobacco control to achieving the Sustainable Development Goals and combat tobacco industry interference in policymaking.^2^ While direct attribution is difficult to establish, four countries that have received investment cases have increased or strengthened their tobacco taxation regime.^2^

Health taxes can be most effective when implemented together, creating a synergistic effect. Alcohol and tobacco are likely complementary goods, meaning that their consumption is correlated; however, the evidence is mixed.^58 59^ If this is the case, increasing the price of cigarettes through taxation could have the side effect of reducing alcohol consumption as well. By extension, increasing the price of alcohol through taxation could further reduce the consumption of cigarettes.

There are several limitations to our analysis. The use of average price elasticity of demand from a sample of eight countries rather than country-specific elasticity may not reflect consumer behaviours in the countries of our analysis. We assumed that the price of the most sold brand of cigarettes is representative of the market and did not incorporate other market segments or account for switching between segments. We also calculated the number of cigarettes sold rather than using primary data and assume consumption is constant between income quintiles, both of which could overestimate the number of cigarettes consumed. Detailed analyses of the impacts of price increases on tobacco use prevalence have been conducted that consider the availability of illicit products, switching to lower price products, temporal differences in elasticity and other factors. For the purpose of the investment cases, however, simplicity and the ability to produce estimates in a timely manner was prioritised to be able to incorporate equity analyses in many of the investment cases. Despite the simple nature of our model necessitated by limited data and the pace of conducting investment cases, our results align with more complex models, which demonstrate additional pro-poor effects of tobacco taxation.^10 11^ The value of the equity analyses lies not in their complexity but in their responsiveness to local needs and their application in moving forward the tobacco control agenda.

## Data Availability

Data sharing not applicable as no datasets generated and/or analysed for this study.
